# Mobile Phone-Based Ecological Momentary Intervention to Reduce Young Adults’ Alcohol Use in the Event: A Three-Armed Randomized Controlled Trial

**DOI:** 10.2196/mhealth.9324

**Published:** 2018-07-20

**Authors:** Cassandra Wright, Paul M Dietze, Paul A Agius, Emmanuel Kuntsche, Michael Livingston, Oliver C Black, Robin Room, Margaret Hellard, Megan SC Lim

**Affiliations:** ^1^ Burnet Institute Melbourne Australia; ^2^ School of Public Health and Preventive Medicine Monash University Melbourne Australia; ^3^ Judith Lumley Centre La Trobe University Melbourne Australia; ^4^ Addiction Switzerland Lausanne Switzerland; ^5^ Behavioural Science Institute Radboud University Nijmegen Netherlands; ^6^ Faculty of Education and Psychology Eötvös Loránd University Budapest Hungary; ^7^ Centre for Alcohol Policy Research La Trobe University Melbourne Australia; ^8^ Department of Clinical Neurosciences Karolinska Institutet Stockholm Sweden; ^9^ Centre for Social Research on Alcohol and Drugs Stockholm University Stockholm Sweden

**Keywords:** alcohol, brief intervention, ecological momentary assessment, randomized controlled trial, mHealth, mobile phone, young adults

## Abstract

**Background:**

Real-time ecological momentary interventions have shown promising effects in domains other than alcohol use; however, only few studies regarding ecological momentary interventions for alcohol use have been conducted thus far. The increasing popularity of smartphones offers new avenues for intervention and innovation in data collection.

**Objective:**

We aimed to test the efficacy of an ecological momentary intervention, comprising mobile Web-based ecological momentary assessments (EMAs) and text messaging (short message service, SMS) brief interventions, delivered during drinking events using participants’ mobile phones.

**Methods:**

We conducted a three-armed randomized controlled trial to assess the effect of a mobile Web-based ecological momentary assessment with texting feedback on self-reported alcohol consumption and alcohol-related harms in young adults. Participants were enrolled from an existing observational cohort study of young adults screened for risky drinking behavior. The intervention group (ecological momentary intervention group) completed repeated ecological momentary assessments during 6 drinking events and received immediate texting-based feedback in response to each ecological momentary assessment. The second group (ecological momentary assessment group) completed ecological momentary assessments without the brief intervention, and the third did not receive any contact during the trial period. Recent peak risky single-occasion drinking was assessed at the baseline and follow-up using telephone interviews. We used a random effects mixed modeling approach using maximum likelihood estimation to provide estimates of differences in mean drinking levels between groups between baseline and 12-week follow-up.

**Results:**

A total of 269 participants were randomized into the 3 groups. The ecological momentary intervention group exhibited a small and nonsignificant increase between baseline and follow-up in (geometric) the mean number of standard drinks consumed at the most recent heavy drinking occasion (mean 12.5 vs 12.7). Both ecological momentary assessment and control groups exhibited a nonsignificant decrease (ecological momentary assessment: mean 13.8 vs 11.8; control: mean 12.3 vs 11.6); these changes did not differ significantly between groups (Wald χ^2^_2_ 1.6; *P*=.437) and the magnitude of the effects of the intervention were markedly small. No other significant differences between groups on measures of alcohol consumption or related harms were observed. The intervention acceptability was high despite the technical problems in delivery.

**Conclusions:**

With a small number of participants, this study showed few effects of an SMS-based brief intervention on peak risky single-occasion drinking. Nevertheless, the study highlights areas for further investigation into the effects of EMI on young adults with heavy alcohol consumption.

**Trial Registration:**

Australian New Zealand Clinical Trials Registry ACTRN12616001323415; https://www.anzctr.org.au/Trial/Registration/TrialReview.aspx?id=369534 (Archived by WebCite at http://www.webcitation.org/7074mqwcs)

## Introduction

Risky single-occasion drinking (RSOD, defined as ≥5 Australian standard drinks, ie, 50 g of alcohol in one session) is a significant cause of preventable morbidity and mortality in Australia and contributes to further social, economic, and legal harms. RSOD is particularly common in young adults; almost half of 18- to 24-year-olds engage in RSOD at least monthly with approximately one-quarter of this age group doing so at least weekly [1]. Similar patterns are observed among 25- to 29-year-olds, with almost 40% engaging in RSOD at least monthly and approximately 20% at least weekly [1].

Brief interventions (BIs) are one of few individual-level strategies that have demonstrated efficacy for reducing alcohol consumption in young people [2,3]; these interventions commonly involve screening and assessing drinking behavior and providing personalized feedback. Research has revealed that BIs can be feasibly and acceptably delivered through Web-based technologies; these innovations reduce cost, enhance convenience, and expand intervention reach. A recent systematic review assessed the effects of 93 computer-delivered interventions and reported small but significant effects on 5 alcohol outcomes [4]. In young people, specifically, Kypri et al [5] and Voogt et al [6] have demonstrated the efficacy of Web-based BIs for reducing RSOD.

Researchers have harnessed mobile phone technology to study alcohol consumption and intervention in participants’ natural environments. Some studies have focused solely on the use of mobile phones as remote data collection tools to conduct ecological momentary assessments (EMA), which are repeated, real-time behavioral surveys. EMAs are an alternative to the usual methods of measuring alcohol consumption, which often involve long-recall periods or averaging of usual drinking [7].

Suffoletto et al used short message service (SMS) text messaging for both data collection and intervention in their studies investigating the use of texting for reducing alcohol consumption [8,9,10]. In a feasibility trial, young adults screened for hazardous alcohol consumption in emergency departments were asked to report the total and maximum single-occasion drinking each week on either Saturday or Sunday via SMS text messaging, with the intervention group sent immediate texting feedback and advice [8]. They found that the intervention group consumed fewer drinks per drinking day in the last month at follow-up compared to controls. In a subsequent study, they expanded their method by asking young people to report their intentions to drink on the coming weekend, commitment to reduce drinking, and later, their actual weekly drinking [9]. Tailored advice was sent to participants based on their responses. The authors found small reductions in their intervention group’s self-reported drinks per day and number of drinking days, compared to the control group [9]. While both previous studies recruited participants from emergency departments, more recent research from Suffoletto et al demonstrated that texting BIs show promise as a booster to a face-to-face-delivered program for reducing alcohol consumption in college students who had violated a campus alcohol policy [10].

Further work has capitalized on the ability for texting to reach participants not only in their environments but also in real-time, as behavior occurs. EMA is a generic term that encapsulates the repeated sampling of behavior in natural environments [11] and has been used to describe both weekly and daily data collection. Kuntsche and Robert demonstrated the feasibility of collecting alcohol EMA data using SMS text message-delivered survey links [12]. In their study, on weekend nights, the participants were sent an SMS text message probing their intention to drink and motivation to drink. The following day, they were asked to report the number of drinks consumed the previous night and whether their drinking had any consequences. Previous research has demonstrated no reactivity to EMAs (ie, completing EMAs does not affect drinking behavior) [7,12].

However, EMA can also be used for event-based sampling, providing rich snapshots into participants’ lives and behaviors as they unfold. EMA with event-based sampling have reduced recall bias, as participants are reporting their behaviors, experiences, and state of mind as they occur, without needing to rely on memory to reconstruct their event [11], thereby facilitating more valid inferences about the nature of time-varying, episodic behaviors, such as drinking, as well as other contextual factors associated with drinking (eg, mood, location, and smoking) [7,11]. When these data are collected together, in real time, we can gain rich and accurate insights into the dynamic patterns of behavior and experience, with an enhanced capacity to detect the antecedents and modifiers of behavior, as well as outcomes [11]. Hence, event-based EMA methods represent an important advancement in our ability to understand alcohol consumption and can be extraordinarily rich sources of data for informing our attempts to modify drinking behavior.

Ecological momentary intervention (EMI) is an extension of EMA that provides intervention based on responses provided in EMA. Logically, the design of EMI depends on the intensity and nature of EMA to which it responds. Just as EMA can be delivered during risk events, such as drinking episodes, EMI can be delivered at a point of time when a behavior of interest is occurring. Cohn et al described the potential for EMI to be delivered when individuals are at greatest risk and when they may be vulnerable to violating a behavior change goal [13]. The effectiveness of real-time interventions appears to be enhanced when EMA are used to tailor content delivered within EMI [14]. In domains other than alcohol use, real-time mobile phone interventions have shown success in improving health behaviors, including sexual health and risk behavior [15,16], smoking cessation [17,18], weight management [19,20], and physical activity [21]. However, few studies have investigated the effects of EMI on alcohol consumption.

Riordan et al focused on tertiary students during the orientation week (the week before classes start for first-year students, usually involving many social events) in their trial of SMS text message-delivered EMI [22]. They sent intervention and control groups 4 EMAs by SMS during orientation week and once per week during semester to assess alcohol consumption in the day prior to assessment. Participants in the intervention condition additionally received an SMS text message with health consequence warnings on each night of the orientation week. The authors reported a reduction in alcohol consumption in females but not in males in the intervention group during orientation week [22]. In a subsequent study of students from 2 residential colleges, Riordan et al sent intervention and control groups 2 EMAs by SMS text messages during orientation week to assess daily alcohol consumption and 7 fortnightly EMAs during the first semester to assess weekend drinking. In addition, an EMI condition comprised 2 intervention SMS text messages per night on 4 drinking-focused social events during orientation week, with content covering social consequences of alcohol use. In one college, a significant difference was found in alcohol consumption with fewer drinks consumed by the EMI condition across orientation week and over the academic year; however, no significant differences were observed between conditions in the second college [23]. A key strength of Riordan et al’s later study is that they began to explore real-time intervention, that is, intervening at the time that the targeted behavior (drinking) was actually occurring. Further opportunities exist to integrate event-based sampling with “real-time EMI;” this involves tracking behavior and responding as it occurs and has proven effective in other health domains [15-21].

This study addresses a gap in this emerging area of research, contributing to the literature on mobile phone-delivered, real-time alcohol EMI. We aimed to test the efficacy of an EMI, comprising mobile Web-based EMAs and texting BIs, delivered during drinking events using participants’ mobile phones. We hypothesized that the EMI group would report a reduction in the alcohol consumption compared with the control group receiving no contact.

## Methods

We conducted a three-armed randomized controlled trial (RCT; ACTRN12616001323415) to assess the effect of a mobile Web-based EMA with texting feedback on self-reported alcohol consumption and alcohol-related harms in young adults. The study was registered with Australian New Zealand Clinical Trials Registration. We adhered to the recommendations of the CONSORT-EHEALTH checklist [24].

### Participants

Participants were recruited from the Young Adults Alcohol Study (YAAS) [25], which is an observational cohort study of young adults living in Melbourne, Australia. This study has included annual telephone interviews, and participants have never previously been offered any intervention. The YAAS cohort study commenced in 2012 with a representative sample of 802 Melburnians aged 18-25 years, screened for engagement in very high-risk drinking (≥7 standard drinks in a single occasion for females and ≥10 for males) [25]. In 2015, the original 802 participants were contacted for a third wave of data collection and invited to participate in the current study. An additional 51 participants were recruited to YAAS in 2015, using the same random digit dialing procedure and screening criteria and were also invited to participate in the current study. Participants were eligible if they owned a smartphone and reported recent risky drinking behavior (≥5 drinks in a single session in the past 3 months). The 2015 YAAS data served as the baseline for this trial, at which time all participants were aged 18-29 years.

### Recruitment and Procedures

Participants who agreed to be contacted about the trial were randomly allocated to one of the 3 arms as follows: an intervention group that received a BI delivered over mobile phone (EMI group) or two control groups in which the participants either completed EMAs without BI (EMA group) or did not receive any contact throughout the trial period (no-contact group). Participants were sent detailed information about their group’s specific procedures and were asked to register to use the relevant intervention. The nature of the intervention meant that it was impossible to blind participants; however, they remained unaware of the detailed procedures of the other arms. Those who did not register for their intervention were followed up by telephone. A non-respondent questionnaire was administered for those contacted to document reasons for refusal. Once registered, participants in the EMI and EMA groups were immediately able to sign up for their “event nights,” which were self-selected nights that they planned to drink on; reminders to sign up for nights were sent weekly for 12 weeks. Follow-up telephone interviews commenced 12 weeks after the first person registered and ran over 4 weeks. Participants were not called until they had reached 12 weeks postregistration but were contacted regardless of their adherence to the intervention (ie, an intention-to-treat approach).

### Design of Ecological Momentary Assessment and Ecological Momentary Intervention

The intervention, including both EMA and intervention message components, was co-designed by young people in a development study that utilized focus group workshops, individual testing, and in-depth follow-up interviews. A total of 40 participants contributed to the development of the overall design of the intervention, including EMA questionnaires, timing, frequency, technology platforms, and message content. After testing the intervention, participants deemed the intervention content, mode of delivery, and burden as acceptable and feasible [26,27]. The content, language, and framing of the content for EMI messages was also informed by the participatory co-design process used in the development study [26], with messages refined according to principles of motivational interviewing theory [28]. We refined our message content based on feedback received in our development study follow-up interviews, which included both a survey, rating scales for individual messages received, and an in-depth interview. This refinement process included removing unpopular messages and message themes, creating alternative messages with similar text for high-rating messages (to ensure variety when tested on multiple nights), and creating new messages as suggested by participants in their follow-up interviews. We then contracted a programmer to build an online module that could capture survey data and send automated tailored SMS text message in response. We spent 4 months testing and refining the system to resolve technical errors before commencing the study.

### Ecological Momentary Assessment Data Collection

Participants from the EMI and EMA groups were asked to choose 6 weekend nights on which they were planning to drink during the 12-week study period to complete EMA surveys. No minimum consumption was specified. Participants were prompted each Thursday afternoon with an SMS text message reminder to register the nights over the weekend on which they planned to drink.

The 6 pm presurvey comprised questions about the participants’ intentions for the night, including how much they planned to drink, spend , and eat; a ranked list of particular adverse events they wished to avoid; their planned mode of transport home; next day plans; any alcohol consumption so far; mood; and the option of writing a message to themselves, which would be sent back to them during the night. At hourly intervals between 7 pm and 2 am, participants were sent a shorter EMA questionnaire asking about the current venue type, alcohol consumption since the last survey, spending, mood, and self-reported drunkenness. Participants were able to opt out of the intervention at the end of each questionnaire if their evening’s drinking was about to end. At 11 am the next day, all participants were sent another questionnaire about alcohol consumption and spending that occurred after the final EMA (ie, after 3 am or when they completed their last EMA of the night), estimated total standard drink consumption and money spent for the night, an estimated volume of water consumed during the night, adverse events, whether a hangover was experienced, and a “fun” rating of the night.

### Ecological Momentary Intervention Messages

In addition to completing EMAs, the EMI group received the texting BI component. Following submission of each EMA questionnaire throughout intervention nights, they received a tailored feedback SMS text message. All feedback SMS text messages during the night contained information reminding the EMI participants of their original intentions or motivations, tips to avoid adverse consequences, or feedback relating to cumulative consumption or spending. These messages were based on a different key variable each hour. The messages comprised a range of columns in which each message was classified by “gender,” “time,” “location,” “drunkenness,” “motivation,” as well as some variables that were pertinent to a particular message type prescribed for a time point (ie, whether the participant had eaten, which was only relevant to tailoring in the first message at 6 pm). Different messages were written to ensure that there was a suitable option for different contexts that they might find themselves in. For example, participants in a nightclub might receive a message reminding them to get water next time they were at the bar, whereas a different message would be suitable for participants at a house party. A range of messages were written for each hour and context based around what participants reported their motivations to be so as to reduce the repetition of messages. The system was set up so that any participant could not receive the same message twice over the course of the whole intervention period. The length of messages varied, with shorter messages sent later in the night, and shorter, simpler messages sent to people reporting higher levels of intoxication. The development of message content for this intervention has been described in two previous publications [27,29], and further detail regarding the tailoring of messages, including examples of messages, are provided in the protocol publication [30]. The messages were underpinned by motivational interviewing and BI theory [26,27]; we used the FRAMES framework to inform our approach [28,31,32].

### Control Groups

The first control group (EMA) followed the EMA data collection procedure described earlier (including registration for 6 intervention nights and all questionnaires on each night) but did not receive any feedback via an SMS text message. This EMA group was included in the trial to investigate reactivity and whether completing assessments alone (without SMS text message feedback) can affect drinking behavior. The second control group (no contact) received no contact until follow-up, which occurred 12 weeks after the baseline assessment. In this study, the no-contact group was the primary control group compared to the EMI group in analysis.

### Reimbursement

Participants from the EMI and EMA groups received reimbursements based on the level of participation in this study. For each event completed (maximum 6), participants received $10. If all 6 were completed, a bonus of $20 was given. Participation in the follow-up survey was valued at $20. Thus, participants who completed all 6 events and the follow-up interview received $100 in cash or voucher. The no-contact group members received $20 for completing the follow-up telephone survey.

### Ethics

We obtained ethics approval for this RCT from the Monash University Human Research Ethics Committee (CF15/3600 - 2015001556). The Alfred Health Research Ethics Committee approved the YAAS cohort study (35/12).

### Primary Outcome Measure

The primary outcome measure was the peak number of drinks consumed in a single night (peak RSOD) at baseline and follow-up, and the primary comparison was between those receiving the intervention (EMI) and the primary control participants (no contact). Our focus on heavy drinking from an occasional or binge perspective is because this pattern is the main risky drinking pattern in our target age group [25,33]. This outcome was measured by asking participants about the number of drinks consumed in their heaviest drinking occasion in the past 3 months at both baseline and follow-up telephone interviews.

### Secondary Outcome Measures

Secondary outcomes of interest were measured at both baseline and follow-up interviews and included alternative measures for risky alcohol consumption and experiences of alcohol-related harms. Secondary alcohol consumption measures were derived from the graduated frequency measures [34]. The graduated frequency questionnaire included the following questions: “In the past 12 months, how often have you had 20 or more standard drinks in a day?” with response options including “Every day,” “5 to 6 days a week,” “3 to 4 days a week,” “1 to 2 days a week,” “1 day a week,” “2 to 3 days a month,” “About 1 day a month,” and “Less often than 1 day a month.” The question was then repeated inquiring about frequency of consumption: 11-19 standard drinks, 7-10 standard drinks, 5-6 standard drinks, 3-4 standard drinks, and 1-2 standard drinks. We used data from these questions to derive annual consumption of >730 standard drinks per year, as it equates to >2 standard drinks per day, which reflects the Australian National Health and Medical Research Council guidelines for alcohol consumption. If >365 drinking days were reported, the 365 heaviest drinking days were included. In addition, we reported on the monthly consumption of ≥11 drinks in a single session. The Australian National Drug Strategy Household Survey commonly uses the threshold of ≥5 drinks in a single session to define risky episodic drinking; however, our participants had already been screened for recent drinking above this threshold and it was therefore appropriate to investigate a higher threshold [25].

Reporting of alcohol-related harms included yes/no/don’t know responses to items derived from the GenACIS [35] and VYADS questionnaires [36], which included the following statements that referred to occurrences of harm on their heaviest drinking occasion in the past 3 months: “Did you get into any verbal arguments or verbal fights on that occasion?,” “Did you fail to do what you intended to do the day after the session?” and “Did you have any trouble getting home on that occasion?” For the transport-related question, respondents answering “yes” were asked to define the nature of the trouble; response options included the following: “Had to wait”; “Didn’t have enough money”; “Missed last train/tram/bus”; “Couldn’t find a taxi/Uber”; “Taxi/Uber wouldn’t take me”; “My lift/designated driver left before me”; “I had an accident (bicycle/other)”; “Got lost”; “Felt unsafe”; “Had to call someone to pick me up”; or “Other (specify).”

Illicit drug use was measured using an item derived from GenACIS [35], which included a Yes/No response to the following statement: “Did you consume any drugs on that occasion that includes illicit drugs, or pharmaceutical drugs that were not prescribed to you?” This outcome was included as reported previously that illicit drug use is associated with heavy drinking events in young people in Melbourne [37].

We assessed both feasibility and acceptability in the follow-up survey using Likert scales to rate several aspects of the respondents’ experiences of the intervention. Both EMA and EMI group participants were asked to what extent they agreed with the following statements: “Filling in the surveys was quick;” “Filling in the surveys was easy;” “I enjoyed filling in the surveys;” “My friends knew that I was doing the surveys during the nights;” “Doing the surveys helped me to think about keeping track of my drinking and spending;” “Doing the surveys helped me to think about having a safer night;” “Doing the surveys didn’t interrupt my night too much;” “I didn’t want friends to know that I was doing the surveys;” “The surveys were too long;” and “Doing the surveys made me want to drink more.”

Furthermore, we asked EMI group participants to evaluate BI message acceptability by asking to what extent they agreed with the following statements: “The messages that I received were useful;” “The messages that I received were relevant;” “I shared the message with my friends during the night;” and “Receiving the messages helped me to keep track of my drinking and spending.”

### Sample Size

Power calculations were based on the primary aim of reducing mean peak RSOD by 2.5 drinks in the EMI group compared to the no-contact group. Assuming an SD of the peak RSOD of 5.2, 67% endpoint participation, 90% power, and 5% significance, we estimated that a sample of 127 participants per group was required. The sample size estimate was calculated to test for a group-by-time interaction from a mixed repeated measures design, and a moderate correlation between subject measurements (*r*=.45, estimated from earlier waves of YAAS data). Further details are reported in the published protocol [30].

### Statistical Analyses

For the primary outcome, we used a random effects mixed modeling approach using maximum likelihood estimation to provide estimates of differences in mean drinking levels between the EMI and no contact group between baseline and 12-week follow-up. In this model, we modeled study participants as random factors (ie, random intercept) and group allocation as a fixed factor. Our primary focus of analysis was the interaction between intervention and study time (baseline vs follow-up). As the sample distribution of participants’ peak RSOD exhibited some evidence of positive skew, we estimated the natural log of the peak RSOD in mixed modeling analyses and reported geometric means. In addition, we used postestimation analyses to derive model marginal means and performed Wald tests for differences between partial interaction terms and their joint significance. Using nested mixed modeling for each group-by-time contrast, Cohen *f*^2^ standardized effect sizes [38], which show the proportion of variance explained for a group, were derived from the decomposition of model residual error terms. To investigate differences between groups in the secondary outcome measures, we used generalized linear mixed modeling specifying a logit link function and binomial distribution. We analyzed data from all participants who consented to participate in the trial and were randomized, regardless of their adherence to the planned intervention and participation in follow-up (ie, an intention-to-treat approach). Maximum likelihood estimation in mixed modeling provided unbiased estimates in the light of study attrition assuming missingness takes a missing at random process [39]. All statistical analyses were performed using the Stata statistical software package (version 13.1, Statacorp LLC, USA) [40].

## Results

### Participants

**Figure 1:**
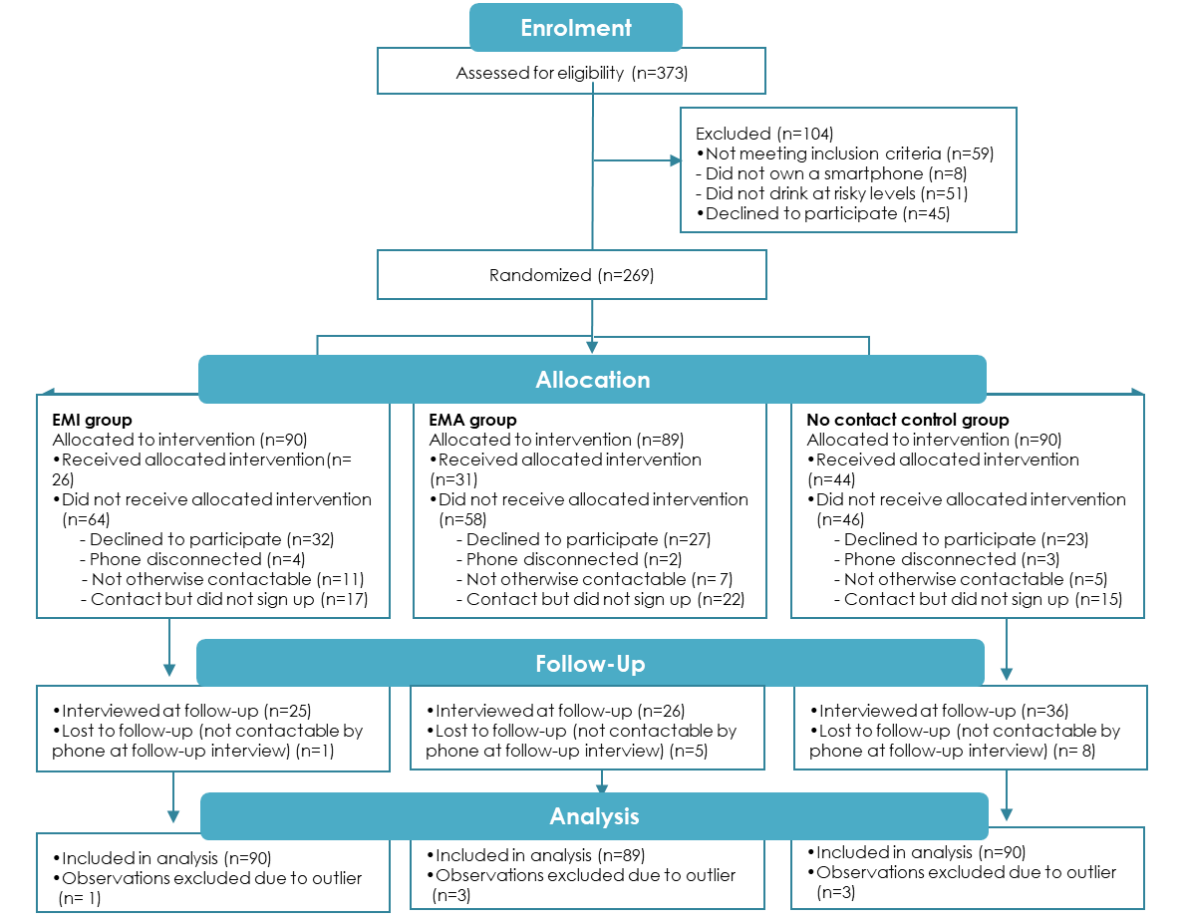
CONSORT flowchart.

Figure 1 presents the participants’ flow. Of the original 802 YAAS participants, 373 completed wave 3 in 2015, which was fewer than anticipated. A total of 59 participants were ineligible for the trial either due to not drinking at risky levels (n=51) or not owning a smartphone (n=8). Of the 314 eligible participants, 269 participants agreed to be contacted about the trial and were randomized into 3 groups as follows: EMI (n=90); EMA (n=89); and no-contact control (n=90). Following receipt of the study information, 101 participants completed the online registration form (EMI=26, EMA=31, and no contact=44), falling short of our target of 300 participants [30]. Of the 81 participants who directly declined to participate over the phone or by SMS text message, the primary reason for refusal was due to work/study commitments (n=23); however, many also stated that they felt that their time in the YAAS cohort meant they had contributed enough to research (n=15). Some participants stated that they did not drink enough for the study to be relevant despite meeting the drinking-related eligibility criteria (n=10). A minority felt that the study design and requirement of 6 intervention nights was too intensive (n=8) or did not provide a reason (n=7). Following the 12-week study period, 87 participants could be contacted for the follow-up telephone survey. Furthermore, 2 participants were excluded from the primary outcome analysis as outliers (SD>3.29 from sample means; ie, *P*<.001) with respect to the peak RSOD measure.

Although rates of registration for the intervention varied between the 3 groups, at intervention commencement there were no statistically significant differences between groups in demographic characteristics (Table 1). The sample that registered to receive the intervention had more females (60%) than males, whereas the 2015 YAAS wave comprised 46% females. There were otherwise no differences in the demographic characteristics of the trial sample compared to participants from the 2015 wave.

**Table 1:** Baseline sample characteristics by study group counts (n), percent (%), and probability values (*P* values) from chi-square inferential tests.

Sociodemographic characteristics			EMI^a^ (n=90), n (%)		EMA^b^ (n=89), n (%)	No contact (n=90), n (%)		Total (n=269), n (%)	*P* value
**Gender**									.38
	Female	46 (51)		37 (42)		45 (50)	128 (48)		
	Male	44 (49)		52 (58)		45 (50)	141(52)		
**Age (n=263)**									.69
	18-24 years	59 (67)		62 (70)		56 (64)	177 (67)		
	25-29 years	33 (29)		26 (30)		31 (36)	86 (33)		
**Country of birth**									.47
	Australia	79 (88)		82 (92)		78 (87)	239 (89)		
	Other country	11 (12)		7 (8)		12 (13)	40 (11)		
**Recreational spending money (Aus $)**									.13
	0-80	9 (10)		16 (18)		6 (7)	31 (12)		
	80-160	24 (27)		21 (24)		16 (18)	61 (23)		
	160-240	17 (19)		16 (18)		27 (30)	60 (22)		
	240+	40 (44)		35 (39)		40 (44)	115 (43)		
	Do not know	0 (0)		1 (1)		1 (1)	2 (1)		
**Currently studying**									.56
	Full-time	24 (27)		27 (30)		30 (33)	81 (30)		
	Part-time	8 (9)		6 (7)		11 (12)	25 (9)		
	Not studying	58 (64)		56 (63)		49 (54)	163 (61)		
**Highest level of education**									.93
	<Year 12	2 (2)		3 (3)		3 (3)	8 (3)		
	Year 12	19 (21)		22 (25)		23 (25)	64 (24)		
	Tertiary	46 (51)		46 (52)		41 (46)	133 (49)		
	Diploma	13 (14)		7 (8)		12 (13)	32 (12)		
	Trade	10 (11)		11 (12)		11 (12)	32 (12)		
**Sexual orientation**									.89
	Heterosexual	80 (89)		79 (89)		81 (90)	240 (89)		
	Bisexual	7 (8)		8 (9)		8 (9)	23 (9)		
	Homosexual	3 (3)		2 (2)		1 (1)	6 (2)		
**Living circumstances**									.14
	With both parents	46 (51)		49 (55)		37 (41)	132 (49)		
	One parent	15 (17)		14 (16)		11 (12)	40 (15)		
	Not with parents	29 (32)		26 (29)		42 (47)	97 (36)		

^a^EMI: ecological momentary interventions.

^b^EMA: ecological momentary assessments.

**Table 2:** Log peak risky single-occasion drinking (RSOD) at baseline and follow-up by study group: marginal geometric means for number of drinks consumed in most recent heavy drinking occasion, *P* value for group by time interaction, and partial group by time contrasts (standardized effect size [Cohen *f*^2^ ] and *P* value) from random effects linear mixed modeling^a^ (n=265).

EMI^b^, marginal means^c^ (CI)		EMA^d^, marginal means^c^ (CI)		No contact, marginal means^c^ (CI)		Group-by-time interaction, *P* value^e^		Partial group-by-time contrasts, *f*^2f^* (P* value)			
Baseline	Follow-up	Baseline	Follow-up	Baseline	Follow-up				EMI vs no contact	EMI vs EMA	EMA vs no contact
12.45 (11.0-13.9)	12.7 (10.2- 15.1)	13.8 (12.1-15.5)	11.8 (9.6-14.1)	12.3 (10.8-13.8)	11.6 (9.7-13.5)		.437		0.001 (.537)	0.02 (.442)	0.01 (.442)

^a^Seven participant observations (EMI, n=1; EMA, n=3; control, n=3) were excluded because of outlying responses (*P*<.001) on the peak RSOD measure.

^b^EMI: ecological momentary interventions.

^c^Geometric means.

^d^EMA: ecological momentary assessments.

^e^Joint Wald test.

^f^Cohen *f*^2^ represents the proportion of variance explained by each group in respective contrasts.

### Participation

For both EMI and EMA groups, 63% (36/57) of participants signed up for 6 or more events and the majority completed surveys for all 6 events (58% [15/26] of the EMI group and 57% [18/31] of the EMA group). EMI participants signed up for a mean of 4.8 events and completed a mean of 4.5 events. EMA participants signed up for a mean of 4.5 events and completed a mean of 4.2. A small number of participants neither signed up for any event (7%; 5/57) nor completed any EMA.

### Outcomes

#### Alcohol Use

Table 2 summarizes results from linear mixed models for the primary outcome analysis. The EMI group showed a small and nonsignificant increase between baseline and follow-up in the mean (geometric) number of standard drinks consumed at the most recent heavy drinking occasion (*M*=12.5 vs *M=* 12.7). The EMA and control groups showed a nonsignificant decrease (EMA: *M*=13.8 vs *M*=11.8; control: *M*=12.3 vs *M*=11.6); these changes did not differ significantly between the groups (Wald *χ*^2^_2_=1.6; *P*=.437) and the magnitude of the effects of the intervention were markedly small [38]. Table 3 shows that no significant differences existed between the groups regarding any other measures of risky alcohol consumption or experiences of harm. For illustrative purposes, the odds ratios presented in Table 3 should be interpreted as follows: the EMI group had 1.4 times higher odds of reporting long-term, high-risk alcohol consumption than the no-contact group (*P*=.76). We performed Ad hoc analyses to include the completion of the 6 “events” with EMI or EMA as a covariate in the model for the primary outcome; however, this did not make any difference in the results (data not shown).

#### Acceptability

Participation rates provide insight into intervention acceptability. Although the primary reasons for nonparticipation were associated with external factors, mostly work and study commitments, the EMI and EMA groups attained lower participation (EMI=26/90, 29%; EMA=31/89, 35%; randomized) than the no-contact group (*n*=44/90, 49%).

Table 4 outlines EMA and message acceptability as evaluated at the follow-up. Most participants in both EMI and EMA groups agreed that the EMA questionnaires were quick and easy to answer. The proportion of participants in the EMI group who reported that they enjoyed completing the questionnaires was higher than that in the EMA group. Participants in the EMI group were significantly more likely to report that their friends knew that they were completing the questionnaires (*P*=.02). In addition, most EMI participants reported that the messages were useful (69%; 17/25) and relevant (88%; 22/25), with just over half agreeing that “receiving the messages helped me to keep track of my drinking and spending.”

Although we tested all features of the intervention comprehensively leading up to implementation, we still encountered problems, including some questionnaire links not being sent on a requested event night, questionnaire links being sent after participants had opted out during an event, and some feedback messages not being sent for EMI participants. Table 5 describes the nature and frequency of technical difficulties encountered during the study period.

### Resourcing Required for Intervention

Both message content and questionnaires were designed during the formative study [26], which took 3 months of a researcher’s time and review from 2 senior researchers. We contracted the Social Research Centre (Melbourne, Australia) to recruit and interview participants. In addition, we purchased a standard license from SurveyGizmo to develop and host our EMA surveys. We contracted SurveySignal to develop and program the online module that sent all EMA SMS text messages, captured data, and sent feedback SMS text message. Researchers spent 4 months testing and refining the program. Each SMS text message cost US $0.10 to send. For each event night, the EMI and EMA groups were sent up to 23 and 13 SMS text messages, respectively. An additional SMS text message per participant was sent to participants in both groups as a reminder each week. Thus, to participate in the intervention as planned with six full events, the total cost of texting was US $15 per EMI participant and US $9 per EMA participant; this does not include participant reimbursement or additional communication regarding technical errors and queries about the research project.

**Table 3:** Secondary measures of risky alcohol consumption and alcohol-related harms: adjusted odds ratio (adjOR), *P* values from group-by-time interactions, and partial group-by-time contrasts from generalized linear mixed modeling (n=269).

Outcome		EMI^a^, adjOR^c^ (CI)	EMA^b^-only, adjOR^c^ (CI)	No contact, adjOR^c^ (CI)	Group-by-time interaction, *P* value^d^	Partial group-by-time contrasts, *P* value		
						EMI vs no contact	EMI vs EMA	EMA vs no contact
**Measures of risky alcohol consumption**								
	High-risk long-term consumption (annual volume ≤730 ASD^e^/year)	1.40 (0.16-12.39)	2.28 (0.27-19.08)	(ref)	.74	0.76	0.75	.45
	Monthly consumption of 11+ drinks in single occasion	4.73 (0.40-40.39)	4.62 (0.55-38.95)	(ref)	.63	0.24	0.31	.16
**Experience of harm on recent heavy drinking event**								
	Any harm	1.43 (0.20-10.07)	0.63 (0.10-4.12)	(ref)	.59	0.72	0.73	.63
	Risk of verbal harm	1.15 (0.03-41.83)	0.74 (0.06-8.97)	(ref)	.57	0.94	0.96	.81
	Risk of transport harm	0.90 (0.03-29.19)	5.86 (0.16-217.58)	(ref)	.32	0.95	0.44	.34
	Failure to complete plans	2.13 (0.23-19.56)	0.94 (0.13-7.01)	(ref)	.96	0.50	0.75	.95
**Use of other drugs on recent heavy drinking event**								
	Use of any illicit drug	1.02 (0.07-15.19)	0.34 (0.02-5.08)	(ref)	.92	0.99	0.69	.43

^a^EMI: ecological momentary interventions.

^b^EMA: ecological momentary assessments.

^c^adjOR shows model interaction term between study group and time and represents the difference in the change in odds (by time) of the outcome between respective intervention groups and the no-contact group.

^d^Joint Wald test.

^e^ASD: Australian Standard Drinks.

**Table 4:** Participant agreement with acceptability-related statements by study group: counts (%) and P values from chi-square inferential tests. Agreement was taken as either “agreeing” or “strongly agreeing” with a respective statement.

Statement		EMI^a^ (n=25), n (%)	EMA^b^ (n=26), n (%)	*P* value
**EMA-related statements**				
	Filling in the surveys was quick	22 (85)	20 (65)	.09
	Filling in the surveys was easy	22 (85)	23 (74)	.34
	I enjoyed filling in the surveys	17 (65)	15 (48)	.20
	My friends knew that I was doing the surveys during the nights	22 (85)	17 (54)	.02
	Doing the surveys helped me to think about keeping track of my drinking and spending	14 (54)	17 (55)	.94
	Doing the surveys helped me to think about having a safer night	14 (54)	12 (39)	.26
	Doing the surveys didn’t interrupt my night too much	19 (73)	15 (48)	.06
	I didn’t want friends to know that I was doing the surveys	0 (0)	0 (0)	—
	The surveys were too long	6 (23)	4 (13)	.31
	Doing the surveys made me want to drink more	1 (4)	1 (3)	.90
**Brief intervention message-related statements**				
	The messages that I received were useful	18 (69)	N/A^c^	N/A
	The messages that I received were relevant	23 (88)	N/A	N/A
	I shared the message with my friends during the night	19 (73)	N/A	N/A
	Receiving the messages helped me to keep track of my drinking and spending	15 (58)	N/A	N/A

^a^EMI: ecological momentary intervention.

^b^EMA: ecological momentary assessment.

^c^N/A: not applicable.

**Table 5:** Participants’ experience of technical difficulty by the study group trial arm: counts (%) and *P* values from chi-square inferential tests.

Technical difficulty type	EMI^a^ (n=25), n (%)	EMA^b^ (n=26), n (%)	*P* value
I tried to sign up for a night but nothing happened at all	13 (52)	14 (54)	.90
I signed up for a night and got a message back to say I registered, but didn’t receive any surveys	11 (44)	11 (42)	.61
I signed up for a night but didn’t receive all surveys	13 (52)	9 (35)	.30
I opted out of the surveys during the night but kept getting surveys through the night	1 (4)	18 (69)	<.001
I had a technical issue actually filling in a survey	9 (36)	1 (4)	.01
I received multiple reminders in one day to sign up for the surveys	15 (60)	15 (58)	.26
The surveys wouldn’t display properly on my phone	4 (16)	0 (0)	.07
I was supposed to get feedback messages but they didn’t come through	5 (20)	N/A	N/A

^a^EMI: ecological momentary intervention.

^b^EMA: ecological momentary assessment.

^c^N/A: not applicable.

## Discussion

### Principal Findings

We tested a novel method to collect data and intervene repeatedly during drinking events. No statistically significant differences in peak RSOD or related harms were detected between the groups. Based on previous EMI and mobile phone-delivered BI studies [10,22,23,41,42], we expected to demonstrate a small but significant effect of our EMI on RSOD compared with our control group. Our development study suggested that our method was acceptable and feasible to participants and demonstrated high levels of engagement and low dropout rate when tested on a single occasion. This study’s finding may be a result of low statistical power, meaning a larger sample is needed in future research to test the effects of our intervention.

Alternative explanations for our null findings may relate to our preselection of heavy drinkers and the potential for regression to the mean [43,44]. The greatest reductions in peak RSOD and alcohol-related harms compared with the no-contact group were observed in the assessment-only group (EMA). Based on previous EMA alcohol studies [7,12] we hypothesized that no reactivity would be observed in this group; however, it is plausible that questions about goal setting and planning in the first questionnaire per night had some intervention effect. These additional questions (recommended by participants in the co-design study for the purpose of message tailoring) require reflection and planning, such as in motivational interviewing therapies [28]. It is possible that responding to these questions could modify RSOD; if this is the case, then it is unknown why the EMA might be more effective without BI messages. The cause could be the specific content of our messages or that BIs were unhelpful in changing alcohol consumption behavior when delivered during drinking events. The latter explanation might be particularly relevant to heavy drinkers; previous research has shown that this population shows increased attentional biases that are thought to promote motivations for alcohol consumption and are resistant to manipulation [45-48]. Thus, research that extends the parameters of our study is needed to assess the effects of EMA and EMI on RSOD, which is important to clarify because if this type of EMA is shown to be effective for changing behavior without BI, then it represents a less intensive and more scalable intervention option, given the complexities involved with effective and acceptable message tailoring. In addition, more participants consented to participate in the EMA than the EMI group, suggesting that the feedback aspect of the EMI was appealing. The EMA group, however, evaluated the questionnaires less favorably than the EMI group and was more likely to drop out of the study between baseline and follow-up, suggesting that the BI messages kept participants more engaged in the intervention.

Overall, our study had lower uptake than we expected, with only 101 participants consenting to participate once they had received the full study information. Our EMI design did require more engagement than previous studies in this area, similar to that by Riordan et al [22,23]; however, we found that only a small proportion of those declining to participate cited study design factors, such as intensiveness and burden, as their reason for refusal (8/81). A far higher proportion reported that they were busy with work or study, not drinking much, or were experiencing research fatigue. From an implementation perspective, there are several other factors, which we believe were important in the small sample size obtained. There was a delay between the YAAS interviews and the RCT information being sent to participants due to the longer time taken to test our texting system, and this may have dulled participants’ enthusiasm. We also found it difficult to clearly and concisely present all information relating to the study in a format that also fulfilled the requirements of the ethics committee. Despite our use of diagrams, the description of procedures seemed overly complex, and we found that when we had the chance to explain the study verbally (in telephone calls to remind/follow-up participants), participants were more inclined to consent. Thus, visual and verbal descriptions of study procedures might be more suitable for complex interventions such as this, where participants are not engaged in-person at any point.

Although this study’s findings were inconclusive, it informed new avenues of investigation for further study into the effects of EMA and BI for reducing alcohol consumption.

### Limitations

This study has several limitations. First, we were unable to recruit the planned sample size; refusal data showed that a major reason was research fatigue due to participants’ prior engagement with the YAAS cohort study. During the recruitment process, few potential participants reported refusal reasons related to intervention intensity or lack of interest; however, the differential recruitment rate between groups suggests that either the intervention lacked appeal or that the study information provided during the consent process was not clear or simple enough. Nonetheless, these findings will inform our future approaches to testing event-level data collection and interventions. Second, challenges related to technical difficulties interfered with the implementation of the EMA questionnaires and feedback messages. Thus, further refinement and testing will be undertaken. Finally, the study relied on self-reported data, which has the potential for reporting bias.

### Conclusions

In conclusion, our study showed no significant differences in peak RSOD, other alcohol consumption measures, or alcohol-related harms between groups of young adults receiving repeated EMAs and EMIs, EMAs alone, or no contact. However, small sample sizes meant that only substantial differences could have reached significance. Our study highlights important considerations for implementing interventions of this type in larger studies. It also identifies directions for further investigation into the effects of EMAs and EMIs on young adults who report heavy alcohol consumption, including the possibility of reactivity to EMAs.

## Abbreviations

BI: brief intervention
EMA: Ecological Momentary Assessment
EMI: Ecological Momentary Intervention
RCT: randomized controlled trial
RSOD: risky single-occasion drinking
SMS: short message service
YAAS: Young Adults Alcohol Study

## Appendix

Multimedia Appendix 1 CONSORT E-HEALTH checklist (V 1.6.1).
